# Immune checkpoint inhibitors-related myocarditis in patients with cancer: an analysis of international spontaneous reporting systems

**DOI:** 10.1186/s12885-020-07741-0

**Published:** 2021-01-07

**Authors:** Rulan Ma, Quanziang Wang, Deyu Meng, Kang Li, Yong Zhang

**Affiliations:** 1 Department of Surgical Oncology, The First Affiliated Hospital of Xi’an Jiaotong University, 277 West Yanta Road, Xi’an, 710061 Shaanxi China; 2 School of Mathematics and Statistics, Xi’an Jiaotong University, 28 Xianning West Road, Xi’an, 710049 Shaanxi China

**Keywords:** Immune checkpoint inhibitors, Myocarditis, Adverse drug reactions, Signal detection

## Abstract

**Background:**

Immune checkpoint inhibitors-induced myocarditis presents unique clinical challenges. Here, we assessed post-marketing safety of cytotoxic T-lymphocyte-associated protein-4 (CTLA-4), programmed cell death-1 (PD-1), and programmed death-ligand 1 (PD-L1) inhibitors by mining the real-world data reported in two international pharmacovigilance databases.

**Methods:**

We analyzed immune checkpoint inhibitors (ICIs)-associated fatal adverse drug events (ADEs) reports from the US Food and Drug Administration (FDA) Adverse Event Reporting System (FAERS) collected from July 1, 2014 to December 31, 2019 and data from EudraVigilance (EV) database accessed on February 29, 2020. Three different data mining approaches were used to detect the signal of fatal myocarditis caused by ICIs.

**Results:**

Based on 7613 ICIs-related ADEs reported to the EV database and 5786 ICIs-associated ADEs submitted to the FAERS database, the most frequently reported ADE was ipilimumab-related colitis. For myocarditis, nivolumab-associated myocarditis was the most common. Among the five fatal toxic effects associated with ICIs, the lethality rate of myocarditis was the highest. Therefore, we further analyzed ICI-associated myocarditis and found that elderly patients and male patients were more likely to develop ICIs-related myocarditis. The results of signal detection showed that the risk signal of avelumab-related myocarditis detected by reporting odds ratio (ROR) method and proportional reporting ratios (PRR) method was the highest, whereas the signal strength of ipilimumab-related myocarditis detected by Bayesian confidence propagation neural networks (BCPNN) method was the strongest.

**Conclusion:**

The findings of this study indicated the potential safety issues of developing myocarditis when using ICIs, which were consistent with the results of previous clinical trials and could provide a reference for clinical workers when using ICIs.

**Supplementary Information:**

The online version contains supplementary material available at 10.1186/s12885-020-07741-0.

## Background

Immune checkpoint inhibitors (ICIs), such as cytotoxic T-lymphocyte-associated protein-4 (CTLA-4), programmed cell death-1 (PD-1), and programmed death-ligand 1 (PD-L1) inhibitors that can inhibit the co-inhibitory immune checkpoint pathways have completely changed the treatment landscape of various malignant tumors [1]. However, with the applications of ICIs increasing, immune-related adverse events (irAEs) associated with ICIs can be induced [2–4]. These irAEs may affect any body system and organ, such as skin, lung, liver, gastrointestinal tract, and so on [5–7]. Though the severe adverse events (AEs) related to ICIs remaining rare, they can be fatal if these side effects cannot be aware by clinicians when using these agents. A systematic review and meta-analysis demonstrated that the fatal ICIs-associated AEs were mainly colitis, hepatitis, pneumonitis, myocarditis, and neurologic effects [5]. Among these fatal toxic effects, myocarditis had the highest fatality rate because it might cause fatal heart failure, arrhythmias, and the like, thus drawing people’s attention to this incident. Another research also highlighted the high mortality rate of severe ICIs-associated myocarditis [8]. However, the published articles about ICIs-related myocarditis are mainly from case reports [9, 10] and clinical trials [11], which usually cannot detect the rare adverse drug reactions (ADRs) and reflect the real risk of ICIs-associated AEs because of inherent limitations of case reports and clinical trials (including limited sample size, missing follow-up information, and the like). Therefore, fatal ICIs-related myocarditis is necessary to be further investigated based on the data from the real-world scenario.

The US Food and Drug Administration (FDA) Adverse Event Reporting System (FAERS) is one of the international spontaneous reporting systems, that is designed to support the FDA’s post-marketing safety surveillance program for drugs approved by the FDA. This database includes all information on adverse drug events (ADEs) and medication errors collected by the FDA. EudraVigilance (EV) is another international spontaneous reporting pharmacovigilance database for ADEs maintained by the European Medicines Agency. Data from these databases can be acquired by the public and utilized to provide evidence for the safe use of the drugs, especially for newly marketed drugs and uncommon ADRs. The objective of this study was to analyze the ICIs-associated myocarditis and to determine the signal of ICIs-associated myocarditis by mining the data reported in the spontaneous reporting systems.

## Methods

### Data sources and processing

Up until now, ICIs approved by FDA for antitumor treatment include pembrolizumab, nivolumab, atezolizumab, avelumab, durvalumab, cemiplimab, and ipilimumab. Therefore, in this study, these seven ICIs were chosen as the study drugs. Spontaneous ADE reports were retrieved from July 1, 2014 (considering the FDA marketing approval of the first ICIs, pembrolizumab on September, 2014) to December 31, 2019 in the FAERS database (https://fis.fda.gov/extensions/FPD-QDE-FAERS/FPD-QDE-FAERS.html), and EV (http://www.adrreports.eu/en/search.html) was accessed and queried on February 29, 2020. In this study, the processing of data downloaded from FAERS database followed the customized strategy described previously [12]. Given that the drug names in the EV and FAERS databases were not standardized, all drug names were standardized into active substances with relevant Anatomical Therapeutic Chemical (ATC) codes before data analysis. Besides, we detected and eliminated duplicates and multiple records (reports with at least overlaps in 3 on 4 of considered key-fields, including event date, age, gender, and reporter country). And the incomplete records with missing event dates, age, gender, or reporter country were removed for further study. Furthermore, all ADEs reported in these databases were coded by preferred terms (PTs) from the Medical Dictionary for Drug Regulatory Activities (MedDRA). Previous studies have demonstrated several fatal ICIs-associated side effects, including myocarditis, colitis, hepatitis, pneumonitis, nephritis, and so on [5, 6, 8]. Therefore, to identify the cases of fatal ICIs-associated ADRs, we searched these spontaneous reporting pharmacovigilance databases using the following MedDRA PTs: myocarditis, colitis, hepatitis, pneumonitis, and nephritis. And these fatal ICIs-associated ADRs were further analyzed in this study by signal-detection algorithms.

Only FAERS, can realize signal detection by using open database, but if you pay for it, other databases can also do it. Therefore, this study used the open database to obtain the number of fatal ICIs-associated ADRs in two major databases, the fatality rate caused by ICIs-associated ADRs in EV database, the age and gender distribution of ICIs-associated myocarditis in EV and FAERS databases, and the signal value of ICIs-associated myocarditis in FAERS database.

### Data mining algorithm

The data mining methods used to detect the ADR signals in spontaneous reporting systems are mainly the disproportionality methods [13, 14], which are based on spontaneous reports submitted for a lot of drugs and ADRs [15]. All the reports included in the FAERS database from July 1, 2014 to December 31, 2019 were selected to determine the ADR signals in the present study.

To detect the ADRs signals, both Frequentist (non-Bayesian) methods and Bayesian methods were used to calculate disproportionality by using reporting odds ratio (ROR) [16], proportional reporting ratios (PRR) [17], and information component (IC) of Bayesian confidence propagation neural networks (BCPNN) [18], which were mainly based on a two-by-two contingency table (Table S1).

PRR and ROR have the advantages of easy calculation and high sensitivity, and the results of PRR and ROR are highly consistent. Therefore, PRR and ROR methods are often used to estimate signals of ADRs. The calculation formulas of ROR and PRR are ROR = (a/c)/(b/d), PRR = a(c + d)/c(a + b), respectively. When an adverse event is new and rare and the second row of two-by-two contingency table involves all drugs (that is, a or b in two-by-two contingency table is a very small number, even zero), PRR and ROR are not applicable. In this scenario, BCPNN can be used to calculate the signal values of ADRs [19]. BCPNN method uses the Bayesian discrimination principle based on the fourfold table. The core of the BCPNN method is to calculate the value of IC. The calculation formula of the IC is IC = ${\log}_2\left(\frac{P_{x,y}}{PxPy}\right)$. Since our study involves some rare ICIs-related ADRs, we use all three data mining methods to determine the signal values at the same time.

For ROR, the threshold criteria of ADR signal are a ≥ 2 and the lower bound of the 95% two-sided confidence interval (CI) is greater than one [12]. For PRR, the signal judgment criteria are a ≥ 3, *x*^2^ ≥ 4, and PRR ≥ 2 [17]. For the IC, the conditions for signal generation are IC > 0 and the lower bound of the 95% two-sided CI > 0 [20]. The higher the value, the stronger the signal appears to be [12, 19].

MATLAB R2019b software was used to detect the ADR signals in this study.

## Results

### Descriptive analysis

**Fig. 1:**
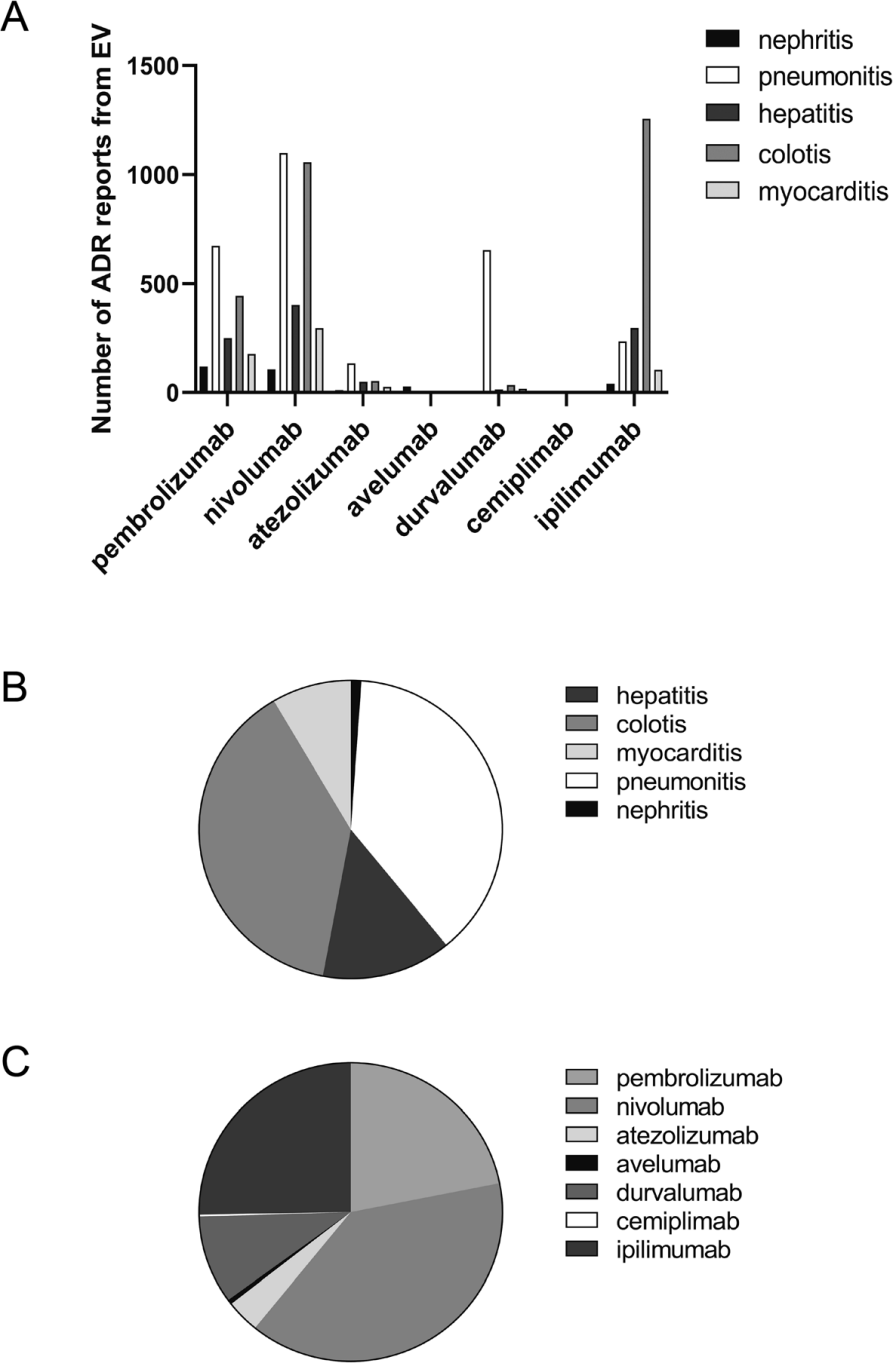
The number of ICIs-associated ADR reports submitted to EV database. **A** The number of ICIs-related ADR reports submitted to EV database. **B, C** The proportion of different ICIs-related ADR reports from EV database. Abbreviations: ADR, adverse drug reaction; EV, EudraVigilance

**Fig. 2:**
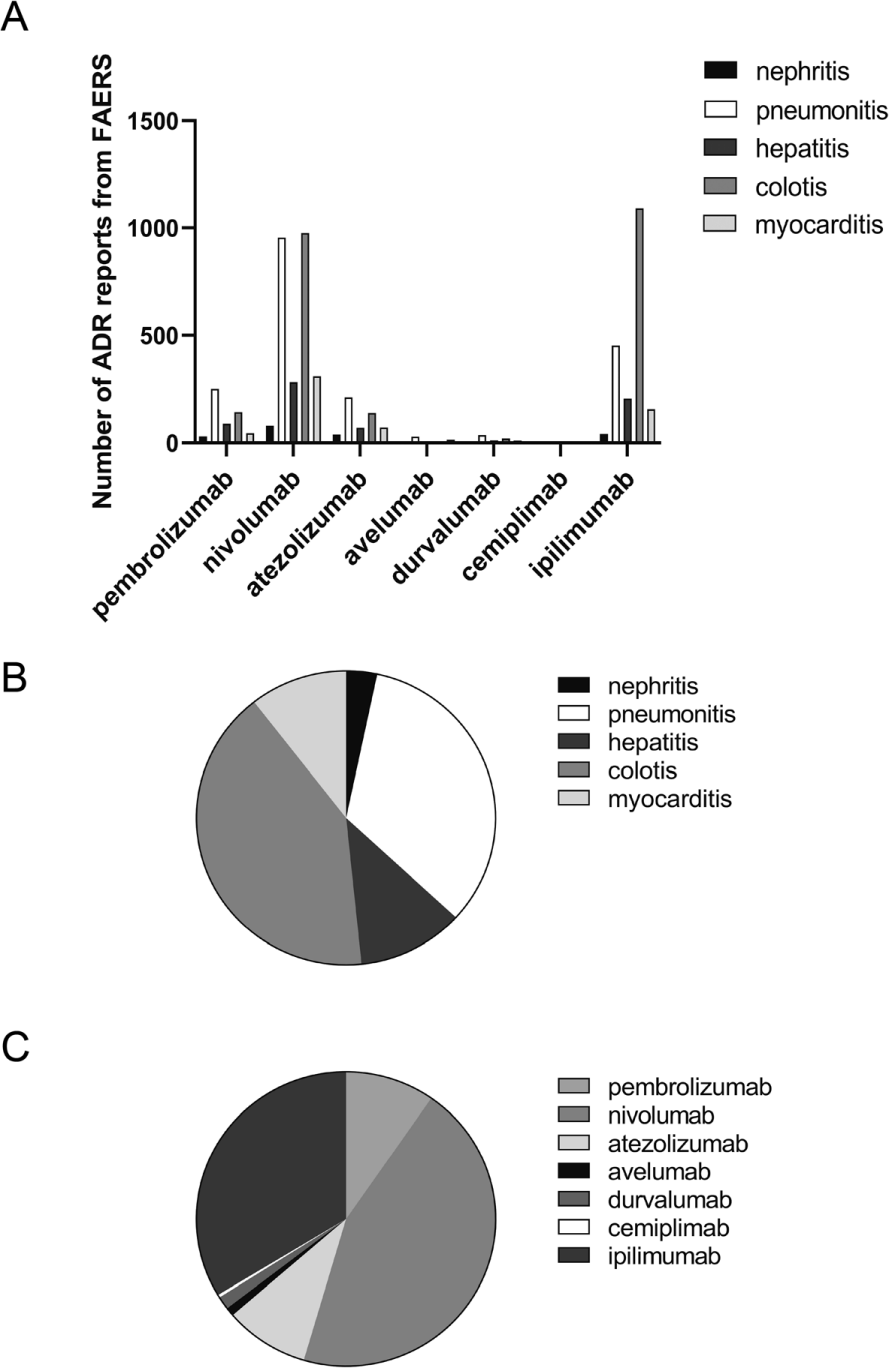
The number of ICIs-associated ADR reports submitted to FAERS database. **A** The number of ICIs-related ADR reports submitted to FAERS database. **B, C** The proportion of different ICIs-related ADR reports from FAERS database. Abbreviations: ADR, adverse drug reaction; FAERS, Food and Drug Administration (FDA) Adverse Event Reporting System

During the study period, a total of 7613 fatal ICIs-associated ADRs were reported to the EV system: 2849 (37.42%) for colitis, 2806(36.85%) for pneumonitis, 1022(13.42%) for hepatitis, 625(8.21%) for myocarditis, and 311(4.09%) for nephritis. The 7613 reports consisted of 2962(38.91%) for nivolumab, 1664(21.86%) for pembrolizumab, 1935(25.24%) for ipilimumab, 725(9.52%) for durvalumab, 272(3.57%) for atezolizumab, 40(0.53%) for avelumab, and 15(0.20%) for cemiplimab (Fig. 1A-C). Besides, the FAERS database received a total of 5786 fatal ICIs-associated toxic effects. The total numbers of ADR cases for colitis, pneumonitis, hepatitis, myocarditis, and nephritis were 2378(41.10%), 1939(33.51%), 666(11.51%), 610(10.54%), and 193(3.34%), respectively. The most frequently reported drug was nivolumab (2599,44.92%), followed by ipilimumab (1946,33.63%), pembrolizumab (558,9.64%), atezolizumab (527,9.11%), durvalumab (83,1.43%), avelumab (55,0.95%), and cemiplimab (18,0.31%) (Fig. 2A-C). We noted that the most frequently reported fatal ICIs-associated ADR was ipilimumab-related colitis, which was 1257 cases of EV database and 1091 cases of the FAERS system. Taken together, among the five fatal ICIs-associated ADRs, colitis caused by ipilimumab was the most common.

### Lethality rates of five fatal ICIs-related ADRs

**Fig. 3:**
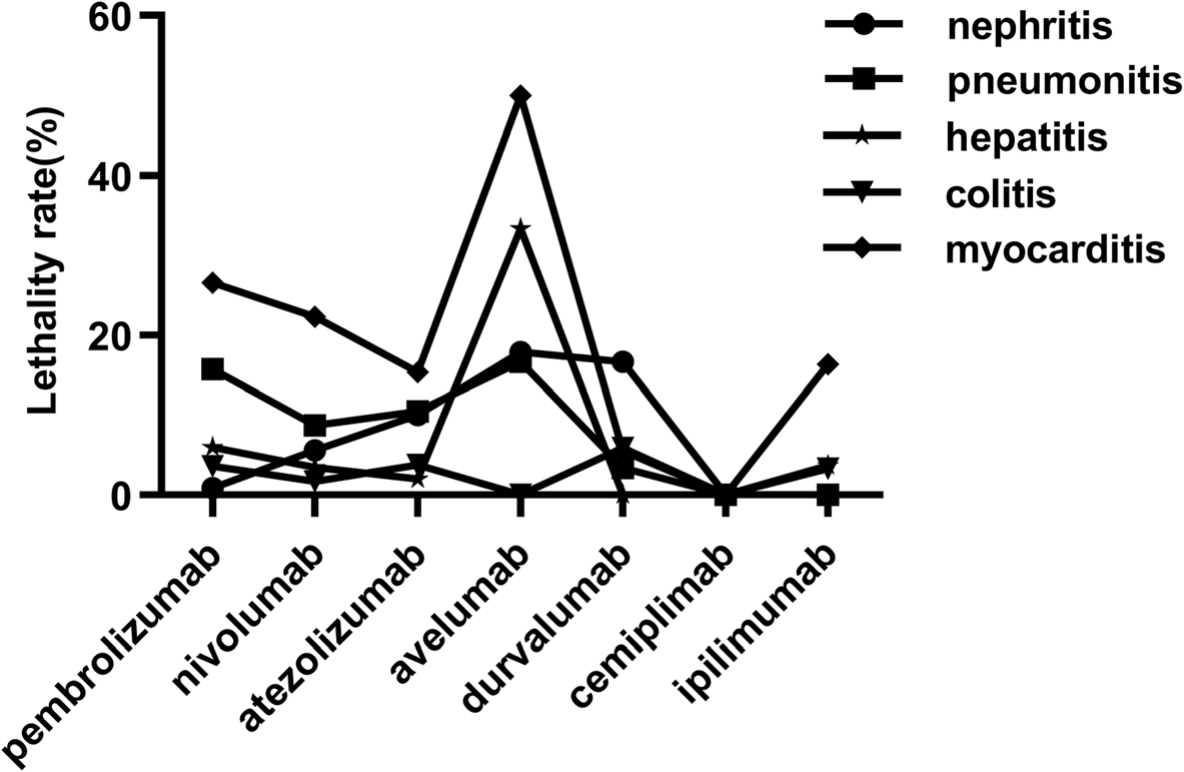
Lethality rates of five fatal ICIs-associated adverse drug reactions

To determine the fatal risk, we measured the lethality rates of five fatal ICIs-related ADRs reported in the EV database. We found that although the number of ICIs-related myocarditis was relatively low, its fatality rate was the highest. The average fatality rate of myocarditis caused by the target drugs was 21,76%. Besides, we noted that the lethality rate of myocarditis caused by avelumab was the highest (50%), followed by pembrolizumab (26.55%). Moreover, although the number of ICIs-related colitis reports was the most, its fatality rate was low. The fatality cases of cemiplimab were 0, which might be related to its short time on the market. To sum up, the fatality rate of myocarditis was the highest, which was consistent with the results of the previous study [5] (Fig. 3).

### Characteristics of the patients with ICIs-related myocarditis

Given that the fatality rate of myocarditis was the highest, we analyzed the characteristics of the patients with ICIs-related myocarditis reported in two spontaneous reporting databases. We found that patients over 64 years old are more likely to suffer ICIs-associated myocarditis, especially aged between 65 and 85 years old. Besides, in terms of gender distribution, male patients were more likely to develop myocarditis than female patients (Table 1).

**Table 1:** Gender and Age Distribution of ICIs-Related Myocarditis

	Pembrolizumab		Nivolumab		Atezolizumab		Avelumab		Durvalumab		Cemiplimab		Ipilimumab	
	EV	FAERS	EV	FAERS	EV	FAERS	EV	FAERS	EV	FAERS	EV	FAERS	EV	FAERS
Total	177	46	296	309	26	70	2	16	18	11	2	3	104	155
Age distribution														
< 18Y	0	0	0	43	0	5	0	0	0	1	0	3	0	20
18-64Y	37	14	55	93	11	33	0	7	3	0	0	0	19	41
65-85Y	96	26	82	170	12	31	2	9	8	5	0	0	28	94
> 85Y	6	6	3	3	0	0	0	0	1	1	0	0	0	0
Unknown	38	0	156	0	3	1	0	0	6	4	2	0	57	0
Gender distribution														
Female	50	15	94	113	12	27	0	5	5	0	0	0	34	53
Male	117	25	188	172	14	40	2	10	11	10	0	2	65	89
Unknown	10	6	14	24	0	3	0	1	2	1	2	1	5	13

Abbreviations: *ICIs* immune checkpoint inhibitors; *EV* EudraVigilance; *FAERS* Food and Drug Administration (FDA) Adverse Event Reporting System

### Signal mining of ICIs-associated myocarditis

**Fig. 4:**
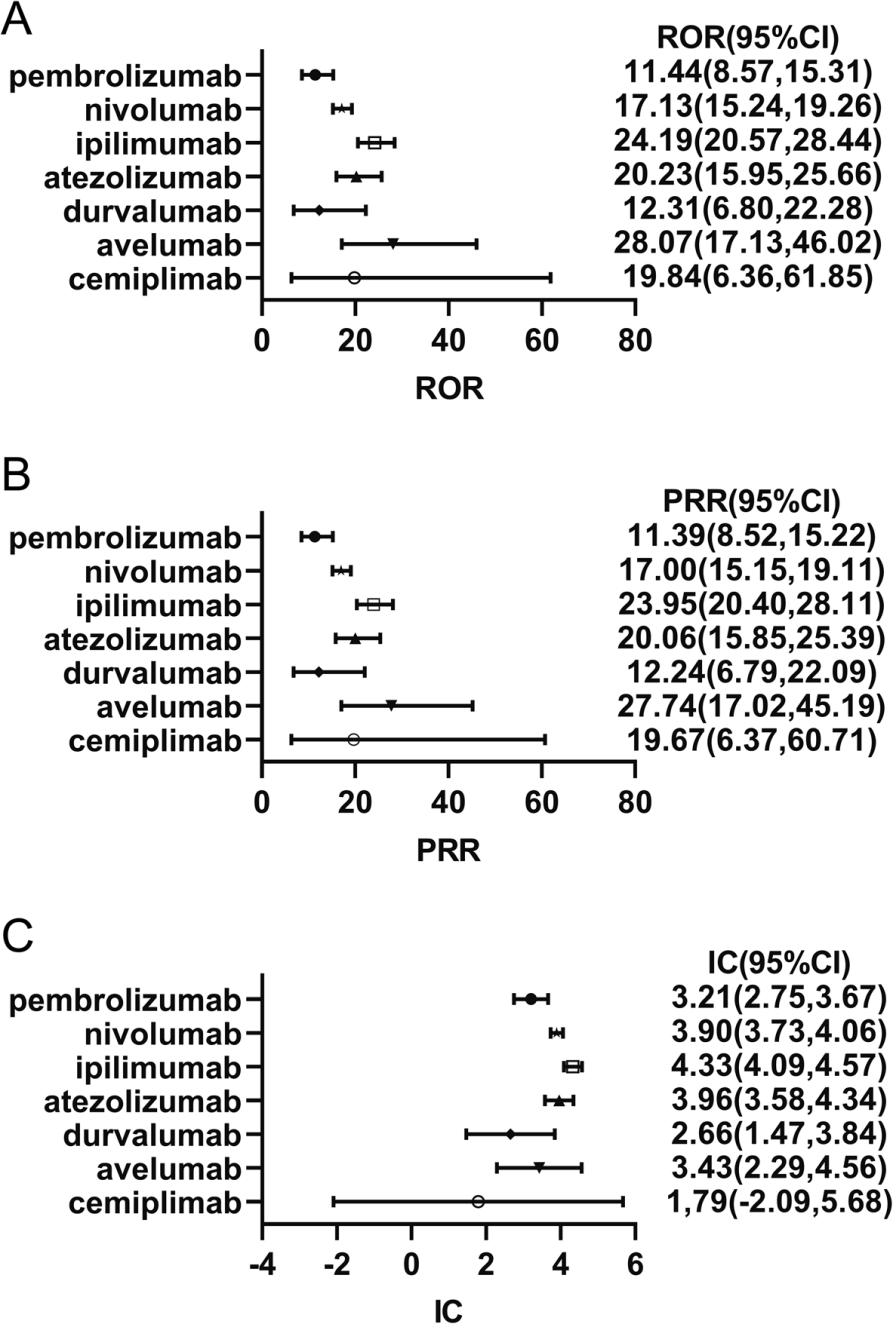
Signal values of ICIs-associated myocarditis. **A** Singal values of ICIs-related myocarditis was detected by using ROR method. **B** Singal values of ICIs-related myocarditis was detected by using PRR method. **C** Singal values of ICIs-related myocarditis was detected by using IC method. Abbreviations: ROR, reporting odds ratio; PRR, proportional reporting ratios; IC, information component; CI, confidence interval

ROR, PRR, and BCNPP methods were used to detect the signal values of myocarditis associated with ICIs in the FAERS database. Similar results emerged by using ROR and PRR methods: the ROR value of myocarditis was 28.07 (95%CI 17.13,46.02) for avelumab, and the PRR for myocarditis was 27.74 (17.02,45.19) for avelumab. The signal value of avelumab-related myocarditis was the highest, whereas the signal value of pembrolizumab-related myocarditis was the weakest [11.44(8.57,15.31) for ROR and 11.39(8.52,15.22) for PRR] (Fig. 4A-B). The highest signal value measured by the IC method was ipilimumab-related myocarditis [4.33(4.09,4.57)] (Fig. 4C). It was worth noting that the IC025 of cemiplimab-related myocarditis was less than 0, so there was no signal generated.

Also, we use the signal mining methods to detect the signal values of the other four fatal ADRs and found that the signal value of ipilimumab-related colitis was the highest, followed by atezolizumab-related nephritis (Table S2).

## Discussion

There is no doubt that immunotherapy based on ICIs are the biggest breakthrough in the field of tumor treatment in recent years, which has brought a satisfactory efficacy for the patients with advanced or refractory tumors and greatly improved the prognosis of the tumor patients. However, the global increase in ICIs use not only brings a satisfactory curative effect for tumor patients but also brings a unique spectrum of fatal toxic side-effects, such as myocarditis, colitis, pneumonitis, hepatitis, and nephritis [6]. Although the risk for mortality of ICIs-related fatal ADRs is lower than common oncologic interventions, clinicians need to raise the awareness of the severity of these toxic effects.

Since human cardiomyocytes express immune checkpoint receptors, ICIs may cause fatal myocarditis while eliminating cancer cells [21]. Several studies reported that the myocarditis associated with PD-1/PD-L1 inhibitors occurred to melanoma patients, which has aroused people’s attention [22–24]. A preclinical research revealed that the left ventricle ejection fraction and global radial strain in transplantable melanoma mice treated with anti-PD-1 antibodies were reduced, compared to the control group, and the analysis of metabolites and lipids indicated dysfunctional energy metabolism, suggesting that immunotherapy based on PD-1 could disturb cardiac function and disrupt cardiomyocyte functional integrity [25]. Javid JJ et al. [8] identified 101 cases of reports submitted to WHO-Vigibase. In these 101 reports of patients with severe myocarditis after treatment with ICIs, most patients (57%) received anti-PD-1 monotherapy, while 27% of patients received anti-PD-1/PD-L1 plus anti-CTLA-4 combination therapy. The results of this study showed that 46 patients died, and the mortality rate of patients with combination therapy was higher than that of patients with monotherapy [8]. Besides, Wang DY et al. [5] retrieved 3545 ADRs reports related to immunosuppressive therapy from 7 academic centers in the WHO-Vigbase database, and systematically reviewed the published researches involved in ICIs. They found that the death of patients treated with anti-PD-1 and anti-CTLA-4 antibodies was usually caused by colitis [32 (37.0%)] and myocarditis [22 (25.0%)], and the fatal toxic effects usually occurred early after therapy initiation for combination therapy. Among these fatal ADRs, the lethality rate of myocarditis was the highest [39.7% (52 of 131 reported cases)]. Furthermore, Mahmood SS et al. [11] created a multicenter registry with 8 sites after observing sporadic cases of ICIs-related myocarditis. 35 patients with ICIs-associated myocarditis were compared with a random sample of 105 patients without myocarditis treated by ICIs. All patients (29% for female patients) were 65 ± 13 years old, and 54% of patients had no other irAEs. The results showed that the prevalence of myocarditis was 1.14% and the median time of onset was 34 days (interquartile range: 21 to 75 days). In addition, a recent review by Raschi et al. [4] summarized several real-world studies (that are focused on irAEs with checkpoint inhibitors) described both rapid (median 16.5 days in combination regimens) and late myocarditis-related events (median 178 days). Taken together, assessing the marketing safety of target drugs by mining the real-world data reported in pharmacovigilance databases is of great significance, and we can fully understand the safety profiles of target drugs by signal detection.

In this study, we analyzed spontaneous reports of several ICIs-related fatal ADRs submitted to EV and FAERS databases and detected signals of ICIs-associated myocarditis by data mining approaches. The findings showed that a total of 7613 fatal ICIs-associated ADEs were reported to the EV database, whereas a total of 5786 ICIs-related fatal toxic effects were submitted to the FAERS database. Among these ADRs, the numbers of ICIs-associated myocarditis were 625 for the EV system, and 610 for the FAERS system, respectively. Although the number of myocarditis reports was not the most, its lethality was the highest, especially the mortality rate of avelumab-related myocarditis was as high as 50%, suggesting that clinicians should take notice of these toxic effects when using immune strategies based on ICIs for tumor patients. We also noted that the most frequently reported ADR was colitis, which was consistent with the results of several systematic reviews [5, 26, 27]. Besides, we found that elderly patients (older than 65-year-old), especially those aged between 65 and 85 years old, were more likely to suffer from myocarditis compared to young patients by analyzing the characteristics of the patients who developed ICIs-associated myocarditis. This could be partially explained by the fact that the immune function of elderly is low and the tumor incidence rate of elderly is higher than young people. Gender distribution of patients with ICIs-related myocarditis showed that male patients were more likely to develop myocarditis compared to female patients, which might be associated with that men play a dominant role in the development of acute myocarditis (sex ratio = 6.75, 8]. And this finding might also be related to the fact that the incidence rate and mortality rate of malignant tumors in males were higher compared to females [28]. Furthermore, the results of signal mining showed that the signal value of avelumab-related myocarditis detected by ROR and PRR methods was the highest, and the highest signal value measured by the IC method was ipilimumab-related myocarditis, suggesting that there had a potential safety issue of myocarditis caused by avelumab or ipilimumab. The fatality cases of cemiplimab-related myocarditis were 0 and there was no signal generated, which might be related to its short time on the market, and further study needs to be explored the risk of cemiplimab-related ADRs.

This study was mainly to analyze the post-marketing safety of ICIs and detected signals of ICIs-associated myocarditis reported in EV and FAERS databases via ROR, PRR, and BCPNN methods. The data of this study were obtained from two international pharmacovigilance databases in the real world, which could provide evidence for the safe use of ICIs to some extent. However, this study has several limitations. First of all, the ADR reports submitted to the EV database and FAERS database usually conclude missing data, duplicate data, the irregular spelling of drug and ADR names, and so on. And most ADRs reports come from America and Europe, while there are few data from Asia or Africa. Secondly, although ROR, PRR, and IC are called quantification signal detection approaches, they are only simple indicators of potential safety issues and cannot quantitatively calculate the incidence (occurrence rates) and the risk of ADRs [4, 13]. Thirdly, although the pharmacovigilance databases are recognized as an important tool to assess the post-marketing safety of drugs by data mining algorithm and the signal detected by data mining methods indicates that the target drug and the target ADR are statistically correlated, it does not mean that the target drug and the target ADR have a biological causal relationship, which needs to be further observed and verified through several large-scale clinical trials [4, 12].

Despite the limitations, the findings of this study indicated the potential safety issues of developing myocarditis when using ICIs, which was consistent with the results of previous clinical trials and could provide a reference for clinical workers when using ICIs.

## Conclusions

The results of this study showed that the application of ICIs was associated with an increase in fatal toxic effects, especially myocarditis, which was consistent with previous studies. It was suggested that clinicians should pay attention to these fatal ICIs-associated ADRs and take preventive measures when treating tumor patients with immunotherapy based on ICIs. The findings of this study also provided objective evidence for post-marketing safety of ICIs, thereby ensuring the safe use of these drugs and improving the prognosis of patients with cancer.

## Supplementary Information

**Additional file 1: Table S1.** A 2 × 2 Contingency Table for Disproportionality Analysis. **Table S2.** Signal Values of ICIs-Associated ADRs.

## Data Availability

All data generated or analyzed during this study are included in this published article and its supplementary information files.

## Abbreviations

ICIs: Immune checkpoint inhibitors
ADRs: Adverse drug events
FDA: Food and Drug Administration
FAERS: FDA Adverse Event Reporting System
EV: EudraVigilance
ROR: Reporting odds ratio
PRR: Proportional reporting ratios
BCPNN: Bayesian confidence propagation neural networks
CTLA-4: Cytotoxic T-lymphocyte-associated protein-4
PD-1: Programmed cell death-1
PD-L1: Programmed death-ligand 1
irAEs: immune-related adverse events
AEs: Adverse events
ADEs: Adverse drug events
PTs: Preferred terms
ATC: Anatomical Therapeutic Chemical
MedDRA: the Medical Dictionary for Drug Regulatory Activities
IC: Information component
CI: Confidence interval

## References

[1] Veronese G, Ammirati E (2019). Differences in clinical presentation and outcome between immune checkpoint inhibitor-associated myocarditis and classical acute myocarditis: same disease, distinct challenges to face. Int J Cardiol.

[2] Postow MA, Sidlow R, Hellmann MD (2018). Immune-related adverse events associated with immune checkpoint blockade. N Engl J Med.

[3] Puzanov I, Diab A, Abdallah K, Bingham CO, Brogdon C, Dadu R, Hamad L, Kim S, Lacouture ME, LeBoeuf NR (2017). Managing toxicities associated with immune checkpoint inhibitors: consensus recommendations from the Society for Immunotherapy of Cancer (SITC) toxicity management working group. J Immunother Cancer.

[4] Raschi E, Gatti M, Gelsomino F, Ardizzoni A, Poluzzi E, De Ponti F (2020). Lessons to be learnt from real-world studies on immune-related adverse events with checkpoint inhibitors: a clinical perspective from Pharmacovigilance. Target Oncol.

[5] Wang DY, Salem JE, Cohen JV, Chandra S, Menzer C, Ye F, Zhao S, Das S, Beckermann KE, Ha L (2018). Fatal toxic effects associated with immune checkpoint inhibitors: a systematic review and meta-analysis. JAMA Oncol.

[6] Boutros C, Tarhini A, Routier E, Lambotte O, Ladurie FL, Carbonnel F, Izzeddine H, Marabelle A, Champiat S, Berdelou A (2016). Safety profiles of anti-CTLA-4 and anti-PD-1 antibodies alone and in combination. Nat Rev Clin Oncol.

[7] Sznol M, Ferrucci PF, Hogg D, Atkins MB, Wolter P, Guidoboni M, Lebbé C, Kirkwood JM, Schachter J, Daniels GA (2017). Pooled analysis safety profile of Nivolumab and Ipilimumab combination therapy in patients with advanced melanoma. J Clin Oncol.

[8] Moslehi JJ, Salem JE, Sosman JA, Lebrun-Vignes B, Johnson DB (2018). Increased reporting of fatal immune checkpoint inhibitor-associated myocarditis. Lancet (London, England).

[9] Chang A, Nasti TH, Khan MK, Parashar S, Kaufman JL, Boise LH, Lonial S, Ahmed R, Nooka AK (2018). Myocarditis with radiotherapy and immunotherapy in multiple myeloma. J Oncol Pract.

[10] Thibault C, Vano Y, Soulat G, Mirabel M (2018). Immune checkpoint inhibitors myocarditis: not all cases are clinically patent. Eur Heart J.

[11] Mahmood SS, Fradley MG, Cohen JV, Nohria A, Reynolds KL, Heinzerling LM, Sullivan RJ, Damrongwatanasuk R, Chen CL, Gupta D (2018). Myocarditis in patients treated with immune checkpoint inhibitors. J Am Coll Cardiol.

[12] Poluzzi E, Raschi E, Piccinni C, De F (2012). Data mining techniques in Pharmacovigilance: analysis of the publicly accessible FDA adverse event reporting system (AERS). Data Mining Applications in Engineering and Medicine.

[13] Hauben M, Zhou X (2003). Quantitative methods in pharmacovigilance: focus on signal detection. Drug Saf.

[14] Zorych I, Madigan D, Ryan P, Bate A (2013). Disproportionality methods for pharmacovigilance in longitudinal observational databases. Stat Methods Med Res.

[15] Michel C, Scosyrev E, Petrin M, Schmouder R (2017). Can disproportionality analysis of post-marketing case reports be used for comparison of drug safety profiles?. Clin Drug Investigation.

[16] van Puijenbroek EP, Bate A, Leufkens HG, Lindquist M, Orre R, Egberts AC (2002). A comparison of measures of disproportionality for signal detection in spontaneous reporting systems for adverse drug reactions. Pharmacoepidemiol Drug Saf.

[17] Evans SJ, Waller PC, Davis S (2001). Use of proportional reporting ratios (PRRs) for signal generation from spontaneous adverse drug reaction reports. Pharmacoepidemiol Drug Saf.

[18] Freitas ABáMLáIREáSOROáALáRMD (1988). A Bayesian neural network method for adverse drug reaction signal generation.

[19] Bate A, Pariente A, Hauben M, Bégaud B. Quantitative signal detection and analysis in pharmacovigilance. In: Andrews E, Moore N, editors. Mann's Pharmacovigilance. London: Wiley; 2014. p. 331–54.

[20] Hauben M (2003). A brief primer on automated signal detection. Ann Pharmacother.

[21] Tarrio ML, Grabie N, Bu DX, Sharpe AH, Lichtman AH (2012). PD-1 protects against inflammation and myocyte damage in T cell-mediated myocarditis. J Immunol.

[22] Johnson DB, Balko JM, Compton ML, Chalkias S, Gorham J, Xu Y, Hicks M, Puzanov I, Alexander MR, Bloomer TL (2016). Fulminant myocarditis with combination immune checkpoint blockade. N Engl J Med.

[23] Heinzerling L, Ott PA, Hodi FS, Husain AN, Tajmir-Riahi A, Tawbi H, Pauschinger M, Gajewski TF, Lipson EJ, Luke JJ (2016). Cardiotoxicity associated with CTLA4 and PD1 blocking immunotherapy. J Immunother Cancer.

[24] Escudier M, Cautela J, Malissen N, Ancedy Y, Orabona M, Pinto J, Monestier S, Grob JJ, Scemama U, Jacquier A (2017). Clinical features, management, and outcomes of immune checkpoint inhibitor-related Cardiotoxicity. Circulation.

[25] Michel L, Hendgen-Cotta UB, Helfrich I, Schadendorf D, Rassaf T, Totzeck M. PD1-blocking immune checkpoint inhibitor therapy for malignant melanoma induces left ventricular dysfunction. Eur Heart J. 2019;40(Supplement_1).

[26] Wei W, Luo Z (2017). Risk of gastrointestinal toxicities with PD-1 inhibitors in cancer patients: a meta-analysis of randomized clinical trials. Medicine (Baltimore).

[27] Wang DY, Ye F, Zhao S, Johnson DB (2017). Incidence of immune checkpoint inhibitor-related colitis in solid tumor patients: a systematic review and meta-analysis. Oncoimmunology.

[28] Bray F, Ferlay J, Soerjomataram I, Siegel RL, Torre LA, Jemal A (2018). GLOBOCAN estimates of incidence and mortality worldwide for 36 cancers in 185 countries. CA Cancer J Clin.

