# Supplementary material for: Immune checkpoint inhibitors-related myocarditis in patients with cancer: an analysis of international spontaneous reporting systems
Source: BMC Cancer. 2021 Jan 7;21:38. doi: 10.1186/s12885-020-07741-0 (PMC7791701; doi:10.1186/s12885-020-07741-0)
Supplement: Supplementary file 1 — Additional file 1: Table S1. A 2 × 2 Contingency Table for Disproportionality Analysis. Table S2. Signal Values of ICIs-Associated ADRs. [file 12885_2020_7741_MOESM1_ESM.docx]

Table S1 A 2×2 Contingency Table for Disproportionality Analysis

| Database | Target AEs | All other AEs | Total |
| --- | --- | --- | --- |
| Target drug | a | b | a+b |
| All other drugs | c | d | c+d |
| Total | a+c | b+d | a+b+c+d |

a: number of reports containing both the target drug and the target adverse event; b: number of reports containing target drug with other adverse events (except the event studied in this research); c: number of reports containing the target adverse drug reaction with other drugs (except the drug studied in this research); d: number of reports containing other drugs and other adverse drug events. Abbreviations: AEs, adverse events.

Table S2 Signal Values of ICIs-Associated ADRs

|  | ROR (95%CI) | PRR (95%CI) | IC (95%CI) |
| --- | --- | --- | --- |
| Nephritis | | | |
| Pembrolizumab | 21.01(14.71,30.02) | 20.94(14.68,28.89) | 3.67(3.02,4.31) |
| Nivolumab | 11.27(8.96,14.17) | 11.25(8.95,14.14) | 3.24(2.91,3.58) |
| Atezolizumab | 30.63(22.26,42.16) | 30.49(22.18,41.89) | 4.11(3.52,4.70) |
| Avelumab | 0.00(0.00,0.00) | 0.00(0.00,0.00) | -0.28(-7.00,6.44) |
| Durvalumab | 9.02(2.90,28.03) | 9.01(2.90,27.94) | 1.58(-1.24,4.41) |
| Cemiplimab | 0.00(0.00,0.00) | 0.00(0.00,0.00) | -0.08(-12.28,12.12) |
| Ipilimumab | 17.35(12.75,23.61) | 17.31(12.73,23.52) | 3.62(3.10,4.13) |
| Pneumonitis | | | |
| Pembrolizumab | 22.02(19.40,25.00) | 21.44(19.85,24.26) | 4.28(4.09,4.47) |
| Nivolumab | 18.39(17.19,19.67) | 18.01(16.86,19.24) | 4.02(3.92,4.11) |
| Atezolizumab | 21.18(18.45,24.32) | 20.64 (18.04,23.61) | 4.21(4.01,4.42) |
| Avelumab | 17.49(12.10,25.29) | 17.12(11.94,24.55) | 3.47(2.82,4.13) |
| Durvalumab | 14.30(10.33,19.81) | 14.06(10.21,19.35) | 3.38(2.84,3.93) |
| Cemiplimab | 9.04(3.37,24.23) | 8.94(3.38,23.69) | 1.79(-0.49,4.06) |
| Ipilimumab | 24.59(22.35,27.05) | 23.87(21.76,26.19) | 4.45(4.31,4.59) |
| Hepatitis | | | |
| Pembrolizumab | 9.50(7.69,11.74) | 9.42(7.64,11.61) | 3.09(2.78,3.41) |
| Nivolumab | 6.40(5.68,7.21) | 6.36(5.65,7.16) | 2.60(2.43,2.77) |
| Atezolizumab | 8.54(6.73,10.83) | 8.47(6.69,10.73) | 2.93(2.57,3.29) |
| Avelumab | 1.50(0.37,5.99) | 1.50(0.37,5.98) | 0.36(-1.80,2.52) |
| Durvalumab | 5.81(3.29,10.25) | 5.78(3.29,10.16) | 2.08(1.12,3.03) |
| Cemiplimab | 23.26(11.53,46.93) | 22.74(11.46,45.11) | 2.73(0.88,4.58) |
| Ipilimumab | 13.64(11.86,15.68) | 13.46(11.73,15.45) | 3.63(3.43,3.84) |
| Colitis | | | |
| Pembrolizumab | 8.90(7.53,10.51) | 8.77(7.44,10.33) | 3.04(2.80,3.29) |
| Nivolumab | 13.46(12.61,14.38) | 13.19(12.36,14.06) | 3.61(2.94,3.44) |
| Atezolizumab | 9.96(8.41,11.79) | 9.80(8.30,11.57) | 3.19(2.94,3.44) |
| Avelumab | 3.47(1.73,6.96) | 3.46(1.73,6.90) | 1.44(0.31,2.57) |
| Durvalumab | 5.61(3.61,8.72) | 5.56(3.60,8.61) | 2.19(1.49,2.89) |
| Cemiplimab | 4.96(1.59,15.44) | 4.92(1.59,15.18) | 1.31(-0.99,3.61) |
| Ipilimumab | 47.08(44.16,50.19) | 43.72(41.18,46.40) | 5.29(5.20,5.38) |

Abbreviations: ICIs, immune checkpoint inhibitors; ADR, adverse drug reaction; ROR, reporting odds ratio; PRR, proportional reporting ratios; IC, information component; CI, confidence interval.
